# Dental Surgeons in the Armies and Navies

**Published:** 1899-09

**Authors:** S. H. Stout

**Affiliations:** Dallas, Texas


For the Texas Medical Journal.

### Dental Surgeons in the Armies and Navies.

BY S. H. STOUT, A. M., M. D., LL. D., DALLAS, TEXAS.

In an interesting article by Herbert H. Shapard, D. D. S., entitled "Dentists in the Army and Navy," which appeared in THE TEXAS MEDICAL JOURNAL in the August (1899) number, I find the following sentence: "I cannot see why it is not just as important to have dentists in the army and navy as to have physicians."

To this I say "Neither do I."

While I was medical director of the general hospitals of the late Confederate army and Department of Tennessee, I directed the employment of dentists from among the detailed men at every hospital post in the department, and experience vindicated me in thus issuing this important direction.

The following narrative is of historical interest, and doubtless Dr. Shapard and his brother dentists will be pleased to read it:

Some time during the winter of 1862-'63, I was called to the dental office of one Dr. Bean (a Marylander by birth, and refugee from Knoxville, Tennessee, the place of his residence, which he left upon the threatened approach of the Federal army, and opened an office in Atlanta, Georgia), to administer chloroform to the wife of a long intimate friend of mine, a captain commissary. Dr. Bean very skillfully and rapidly extracted more than a dozen diseased teeth from the lady's mouth. He had a full equipment of instruments, was well educated, and skillful. He was suffering from chronic rheumatism, some of the joints of his lower extremities much distorted. I complimented him upon his skill. He proposed to appoint two days in the week to do dental work for the soldiers in hospitals free of charge. His proposition I cheerfully accepted, and issued a circular to all the surgeons in charge of the hospitals in Atlanta urging them to avail themselves of the offer of Dr. Bean, and also to freely admit his presence at any and all surgical operations he wished to see. He was a gentleman of far more than mediocre intelligence, as well as mechanical skill. While visiting the

Medical College Hospital, Dr. Bean's attention was called by Surgeon W. F. Westmoreland, in charge of that hospital, to the difficulty of making patients comfortable when suffering from gunshot fractures of the lower jawbone. Dr. Bean, at his request, devised an appliance made of wire which was comfortable to the patients and permitted them to talk, and even in many cases to masticate their food. So great was the success of Dr. Bean's apparatus for the treatment of fractures of the lower jaw, that I directed all such cases to be sent to the Medical College Hospital that they might have the benefit of treatment under Surgeon Westmoreland and Dr. Bean.

Surgeon Covey (formerly of the U. S. Army) being sent to the Department of Tennessee by the Surgeon General, S. P. Moore, on a tour of inspection, was so pleased with the appliance invented by Dr. Bean that he made it the subject of a special report, which was published in the *Confederate States Medical Journal,* of which I now have a copy.

By a special order of the Surgeon General, Dr. Bean went to Richmond and there demonstrated his appliance to the surgeons on duty in the hospitals there. A few years after the Confederate war Dr. Bean visited Europe, and while exploring the Alps he was frozen to death. He was a man of liberal education, a scientific and most skillful dental surgeon, worthy of honorable remembrance by all the members of his useful and progressive profession. Peace to his ashes.

In my administration as hospital director, I early directed the purchase of dental materials and instruments with the hospital funds, to be used by dentists detailed for that service in the hospitals, under the supervision of the surgeons. After, on several occasions, pressing the subject upon the attention of the Surgeon General, he issued a circular authorizing the employment of dentists of qualifications approved and vouched for by the medical directors, with the rank and pay of hospital stewards. A form of monthly reports of the work of these dentist hospital stewards was prepared and forwarded to the medical directors, with the approval of the surgeons in immediate charge of the hospitals. The dentists made monthly reports from which were made consolidated reports that were forwarded monthly by the medical directors to the Surgeon General. The cost of dental materials and appliances was paid for out of hospital funds. I have in my possession now, copies of some of those printed forms of dental reports.

Thus you will see that I am the first army medical officer in high authority, who ever recognized the importance of the services of dental surgeons, and the first to use them in army practice. Had the Confederacy survived the conflict of arms with the U. S. government, I doubt not the regulations of its army and navy would have contained provisions for the utilization of the services of dentists, in both field and hospital service and aboard war vessels.

The facts above stated were of general notoriety during and for several years after the Confederate war. The now venerable W. H. Morgan, M. D., D. D. S., Professor in the Dental Department of Vanderbilt University, early became cognizant of them, and at one of the earlier meetings of the Southern Dental Association nominated me and secured my election as an honorary member thereof.

Just why it is, after such a signal vindication of the practice of employing dental surgeons in army practice, the practice has not been adopted in the U. S. armies and navy, we are left to surmise. Is it because the officials of those military branches of the public service are so wedded to the old regulations, which are a century or more old, and which in many particulars, are not appropriate to field service in time of war, nor in line with the progress of the age, that they cannot get out of the ruts that prevent the effective care of the sick and wounded, because of the routine usages of military establishments, which in times of peace move and act slowly on account of the red tape that often obstructs rather than helps active movements, and, because of the delays it causes, inflicts great hardships upon suffering soldiers?

It is a fact of record that the results of medical and surgical practice in the Confederate armies were numerically more favorable than those achieved by the medical staff of the Federal armies, although the former labored under the disadvantage of a meager supply of medicines and surgical appliances, which, by the Federal government, were declared to be contraband of war. It would perhaps be deemed uncharitable to surmise that those responsible for the care of the soldiers and sailors of the United States, when sick and wounded, like the Jews of old, who believed that "no good could come out of Nazareth," think no good lessons touching the care of the sick and wounded in time of war can be learned from the experiences and successes of the medical officers in the service of the Confederate armies and navy.

